# Rapid increase in abundance and distribution of invasive pink salmon (*Oncorhynchus gorbuscha*) within a diverse, large Barents Sea catchment

**DOI:** 10.1111/jfb.70348

**Published:** 2026-02-11

**Authors:** Jaakko Erkinaro, Panu Orell, Frode Fossøy, Mikko Kytökorpi, Karl Gjelland, Narve Johansen, Sigurd Domaas, Jorma Kuusela, Pierre Fagard, Eirik Frøiland, Morten Falkegård

**Affiliations:** ^1^ Natural Resources Institute Finland Teno Fisheries Research Station Utsjoki Finland; ^2^ Norwegian Institute for Nature Research Centre for Biodiversity Genetics Trondheim Norway; ^3^ Norwegian Institute for Nature Research FRAM – High North Research Centre on Climate and the Environment Tromsø Norway; ^4^ Tanavassdragets fiskeforvaltning Tana Norway; ^5^ County Governor of Troms and Finnmark Vadsø Norway

**Keywords:** alien species, Atlantic Ocean, eDNA, expansion, pink salmon, watershed

## Abstract

Pink salmon originate from the North Pacific area but were introduced into northwest Russia from the late 1950s onwards. Since 2017, the alien species has increased dramatically in abundance and rapidly invaded adjacent areas of the North Atlantic region. In the large Teno River in northernmost Norway and Finland, running to the Barents Sea, various monitoring methods originally designed for assessment of Atlantic salmon populations have been used to observe the development in abundance and distribution of pink salmon in the main stem and in a number of tributaries. In addition, environmental DNA (eDNA) sampling has enabled monitoring of these trends across an even wider set of tributaries. The first observations of pink salmon were made in the 1960s, and variable but mostly low catches were recorded in the following decades. In recent years, the total number of pink salmon entering the Teno system increased rapidly from c. 5000 in 2017 to c. 180,000 in 2023. Initially, the invading pink salmon were occupying the main stem, large tributaries and headwater rivers of the catchment, even up to a distance of 250–350 km from the sea. However, in recent years, a greater number of smaller tributaries have been occupied as demonstrated by eDNA detections and other observations. The largest spawning aggregations of pink salmon have been observed in the main stem of the Teno River. Future development in the abundance and dispersal of pink salmon in the Teno system depends strongly on the extent and success of the mitigation efforts in intercepting and removing pink salmon by a weir and trap close to the estuary.

## 1 INTRODUCTION

Pink salmon (*Oncorhynchus gorbuscha* Walbaum) is the smallest and most abundant of the five semelparous salmon species of the genus *Oncorhynchus*, native to the North Pacific area (Quinn, 2018). The species was introduced to the Atlantic area, in the Russian White Sea basin, in the late 1950s (Alekseev et al., 2019; Sandlund et al., 2019). Between 1956 and 1979, more than 220 million pink salmon eggs and fry from the southern part of Sakhalin Island in the Pacific Ocean were transported to rivers and local hatcheries in the White Sea basin. These introductions resulted in variable but in some years relatively abundant returns and catches of adult pink salmon which were dependent on stocking activity only, and the original aim of establishing permanent self‐sustaining populations was not reached (Sandlund et al., 2019). However, after the change of the donor population to a more northerly one from the Ola River, a North Okhotsk Sea drainage in the Magadan oblast, in 1985, naturally spawning populations were gradually established in the White and Barents Sea rivers of Russia and also in northeastern Norway (Gordeeva et al., 2015; Niemelä et al., 2016; Sandlund et al., 2019). Successfully established, self‐reproducing, odd‐year populations in the Barents and White Sea rivers are now spanning almost 20 successive generations and the even‐year population in the North Atlantic area has, to date, proven clearly less successful than the odd‐year population (NHPSEG, 2023).

After the change in donor stock, pink salmon catches in northwestern Russia have generally increased in the 2000s, but after a slow, stepwise development, catches showed an abrupt, increase in 2017 (NASCO, 2024). Also, in other parts of the northeastern Atlantic area, after three decades of slow development of pink salmon abundance since the mid‐1980s, a sudden and widespread increase in the distribution and abundance of pink salmon started in 2017. Since then, pink salmon have rapidly spread from the Barents and White Sea across a wide area in the North Atlantic (ICES, 2024; NHPSEG, 2023; Sandlund et al., 2019). This population expansion was characterised by markedly increased abundances in the Barents Sea area in 2017 and 2019, followed by even higher runs of fish in 2021 and 2023, especially in Norway (NASCO, 2024; Staveley, Ahlbeck‐Bergendahl, et al., 2025). The abundance of pink salmon has massively increased, especially in the Nordic countries, where 50 times more were recorded in 2023 than in 2017 (Staveley, Ahlbeck‐Bergendahl, et al., 2025). In Russia, however, markedly fewer fish returned in 2023 than in 2021 (NASCO, 2024). Despite the increase in distribution since 2017, pink salmon abundance has remained much lower further south in Western Europe, Iceland, Greenland and Eastern North America compared to the Barents Sea area (ICES, 2024; NASCO, 2024; Staveley, Ahlbeck‐Bergendahl, et al., 2025).

The sudden increase in abundance and distribution of pink salmon in the North Atlantic area has raised concern and uncertainty about how the rapidly spreading alien species will affect native ecosystems and especially valuable Atlantic salmon (*Salmo salar* L.) populations (e.g. Hindar et al., 2020). In the Pacific region, strongly increasing numbers of pink salmon can have adverse effects on other species, broadly throughout the ecosystem (Ruggerone et al., 2023) and, for instance, on Chinook salmon (*Oncorhynchus tshawytscha* Walbaum), a species of the same genus (Ruggerone et al., 2025). However, the effects of the recent pink salmon invasion on the native species and ecosystems in rivers of the North Atlantic and Barents Sea region are currently largely unknown (Dunmall et al., 2025; Lennox et al., 2023; NHPSEG, 2023). An additional concern has been whether increasing numbers of pink salmon affect the value of recreational fisheries in North Atlantic rivers and modify the behaviour and preferences of anglers (Guay et al., 2023). As a proactive mitigation measure in odd years, various removal fishing methods have been tested and operated across northern Norwegian salmon rivers since 2017 (e.g. Anon., 2021). In 2023, stronger and more coordinated efforts were put in place, and removal fishing for pink salmon, using traps, seines, gill nets etc., was operational in a number of Norwegian rivers (National Expert Group for Measures Against Pink Salmon 2024). Thorstad et al. (2025) assessed the effect of different removal methods in 94 rivers in Norway. The results showed that bank‐to‐bank traps can be constructed to prevent pink salmon from entering even relatively large rivers, with immediate mortality of native salmonids as low as 0.1%–0.5%. However, some of the traps included in this study had low efficiency in catching pink salmon. In the largest of the northern Norwegian salmon rivers, the Teno/Tana River, a trap with long guiding fences captured less than 10% of ascending pink salmon, and in contrast, upstream Atlantic salmon migration was delayed for varying periods (Domaas et al., 2024). For facilitating migration of the native species, occasional openings were made in the guiding fences (Domaas et al., 2024). The effects of these strong measures, especially long‐term impacts on pink salmon runs and native migratory salmonids, are yet to be analysed (e.g. Berntsen & Havn, 2024; Domaas et al., 2024; Dunmall et al., 2025; Thorstad et al., 2025).

In the new and expanding distribution in the Atlantic area, pink salmon abundance and distribution have been documented at a country/jurisdiction or catchment scale (Berntsen & Havn, 2024; ICES, 2024; NASCO, 2024; Staveley, Ahlbeck‐Bergendahl, et al., 2025) but little or no distribution data have been published within catchments at a tributary scale. Monitoring of the native Atlantic salmon populations takes place in a number of locations within the large Teno River system in northernmost Finland and Norway where, in addition to the sonar monitoring of ascending fish in the main stem, time series of fish counts are also available for tributary‐specific stock assessment (Anon., 2025). The monitoring network in the Teno catchment also provides a unique opportunity to investigate the pink salmon invasion at a tributary scale throughout this large subarctic river system. The aim of this study is to use data from various monitoring programs and catch statistics and environmental DNA (eDNA) surveys to provide an overview of the development of the pink salmon invasion, their occurrence, distribution and abundance across different parts of the Teno River catchment.

## 2 MATERIALS AND METHODS

### 2.1 Study area

**FIGURE 1:**
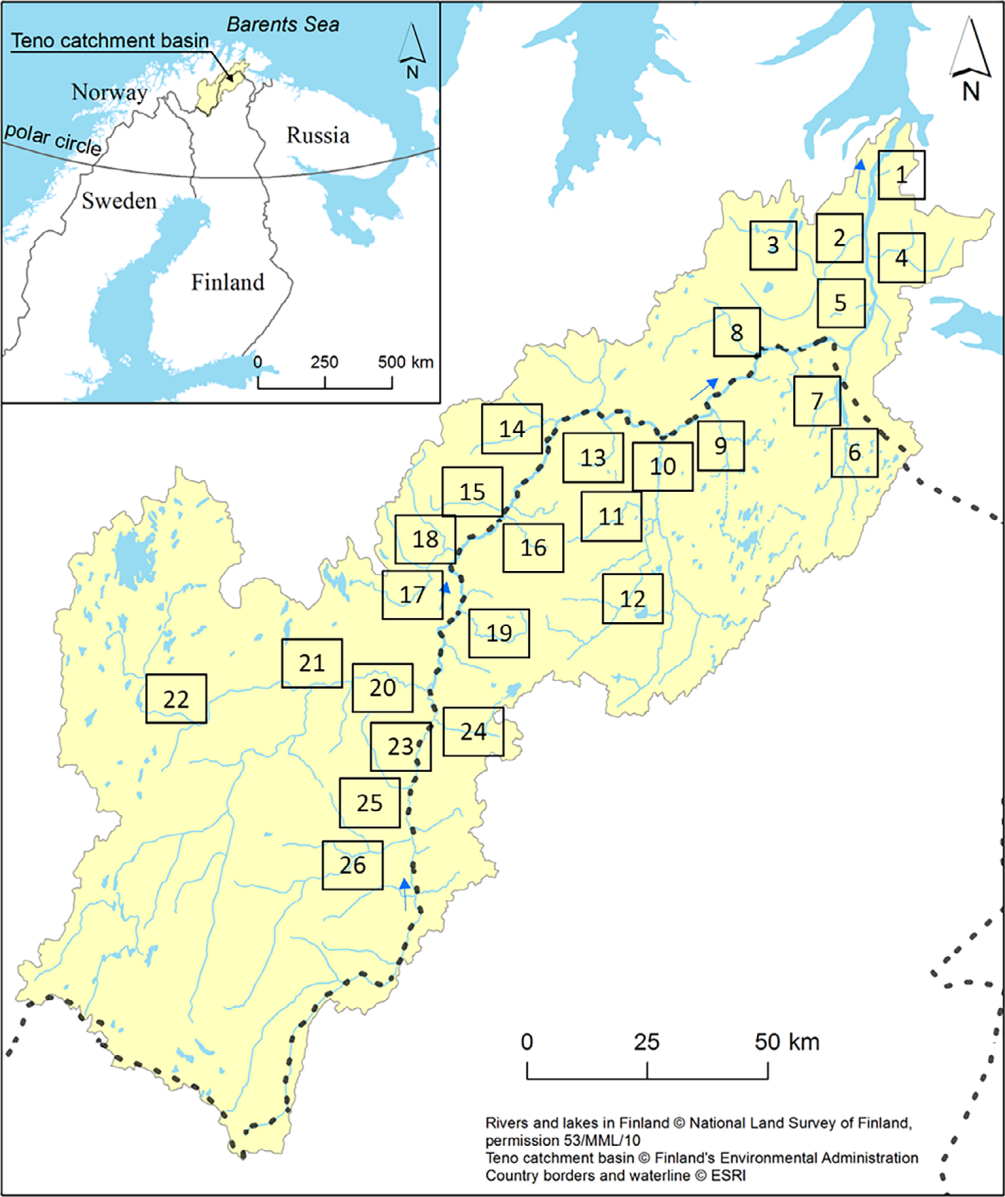
The Teno River catchment and tributaries where occurrence of pink salmon has been monitored. For the names of numbered tributaries, see Table 1.

The Teno River (Norwegian: Tana, Sámi: Deatnu) forms the border between northern Finland and Norway (68–70°N, 25–27°E) and drains into the Tanafjord at the Barents Sea coast (Figure 1). More than 1100 km of the different stretches of the system, including the main stem, the large headwater branches and numerous smaller tributaries, is accessible to anadromous salmonids (Figure 1). The river is free‐flowing and drains a near‐pristine, subarctic catchment with relatively little human disturbance. The mean annual discharge is 170 m^3^ s^−1^, with high seasonal variability: spring runoffs may reach 1500–3000 m^3^ s^−1^.

The Teno River is a major Atlantic salmon catchment supporting an extremely diverse population complex consisting of more than 20 demographically independent, genetically distinct and temporally stable population segments in various tributaries and different parts of the main stem (Vähä et al., 2008, 2017). In addition, the life‐history variation of Atlantic salmon in this system is one of the most diverse documented (Erkinaro et al., 2019). Until recent years, the Teno River has also supported various forms of recreational and net fisheries, which have yielded large annual freshwater catches between 80 and 250 metric tonnes, or 20,000–60,000 individual Atlantic salmon (Erkinaro et al., 2019; Niemelä et al., 2006). However, the recent steep decline in salmon stock status has resulted in a ban on salmon fishing since 2021 (Anon., 2025).

Atlantic salmon stocks and their fisheries within the Teno system are regulated by bilateral agreements between Finland and Norway, with the aim of conserving the wild populations and also supporting sustainable fisheries with special emphasis on the traditional fishing culture of the indigenous Sámi people (Hiedanpää et al., 2020). Supplementation of wild populations through the stocking of reared fish or eggs is also strictly forbidden in the Teno system.

Like the Barents Sea rivers in general, the Teno River has been subject to the pink salmon invasion since the early 1960s, and since 2017 in particular (Niemelä et al., 2016; Sandlund et al., 2019), experiencing rapidly growing numbers in odd years (ICES, 2022). Pink salmon have reproduced successfully and increasingly in recent years (Erkinaro et al., 2022; Erkinaro & Orell, 2022) and juvenile production has been documented in different parts of the river system (Erkinaro et al., 2024; Pohjanheimo, 2025).

### 2.2 Fish counts and catches

Pink salmon run sizes have been monitored using the same methods used in Atlantic salmon monitoring programs within the Teno system. This includes counting the number of ascending adult fish using sonars and video arrays, spawner counts by snorkelling and catch information (Anon., 2025).

The longest time‐series of migratory fish count data are available from a video camera array including eight cameras installed in the Ohcejohka (Utsjoki), a large tributary on the Finnish side of the Teno catchment (Table 1 and Figure 1). These cameras have counted upstream and downstream migrating fish at this site since 2002 (Orell et al., 2007). Another video array at Lákšjohka has been operational in 2009–2020, and in a few other tributaries video monitoring has been trialled in some years (Table 1.). In addition, varying numbers of video cameras have been used at all sonar monitoring sites to facilitate fish species identification and validate information collected by the sonar arrays (Anon., 2025; Pohjola et al., 2020; Räty et al., 2025). Back and forth swimming behaviour of pink salmon at the video arrays of some tributaries has been a challenge in estimating the run sizes in some years; however, the net numbers of fish (ascending – descending) have been used as the yearly population estimate (Table 1).

**TABLE 1:** Pink salmon monitoring and observations in tributaries of the Teno River catchment.

#	Tributary	Method	2005	2006	2007	2008	2009	2010	2011	2012	2013	2014	2015	2016	2017	2018	2019	2020	2021	2022	2023	2024
1	Hárrejohka	E	**‐**	**‐**	**‐**	**‐**	**‐**	**‐**	**‐**	**‐**	**‐**	**‐**	**‐**	**‐**	**‐**	**‐**	**‐**		X	X	X	X
2	Máskejohka	T																	490^a^		2567	
3	Geasis	D																			0	0
4	Luovtejohka	D									0						0				647	1
5	Lišmmajohka	O																			100 s	
6	Buolbmátjohka	D	0	0	0	0	0	0	0	0	0	0	0	0	0	0	0	0	2^b^	0	0	0
7	Gálddašjohka	V																			3	0
8	Lákšjohka	V/D					2	0	0	0	1	1	1	2	25	3	3	2			0^c^	0^c^
9	Veahčajohka	V												3					380			2
10	Ohcejohka	V	7	3	3	2	2	0	1	4	0	7	2	5	50	3	27	6	473	4	653	1
11	Čársejohka	E																	X	X	X	X
12	Geavvu	E																	X	X	X	X
13	Goahppelašjohka	E															X		X	X	X	X
14	Leavvajohka	D		0													0				23	0
15	Báišjohka	D		0								0	0	0	0	0					50	0
16	Njiljjohka	D		0			0	0	0	0		0	0	0	0	0		0	0	0	0	
17	Váljohka	V											0									
18	Ástejohka	D											0						0		0	0
19	Áhkojohka	D	0	0	0/0^d^	0	0	0	0	0	0	0	0	0	0	0	0	0	0	0	0	0
20	Kárášjohka	S/V														0	143	0	948	0	133	1 s
21	Geaimmejohka	D																			2	0
22	Iešjohka	S/V															0	0		0		1 s
23	Anárjohka	S/V			1^b^			1^b^					7^b^	3^b^	11^b^	12	353	1^b^	1320		3805	13^b^
24	Gáregasjohka	V														0						
25	Iškorasjohka	D																			0	
26	Goššjohka	E																	X	X	X	X

*Note*: The numbering of tributaries follows the locations indicated in Figure 1. Main tributaries in bold typeface, and below, secondary tributaries running to the main tributaries in light typeface and indented. Observations in numbers of fish or in abundance categories (1, 10, 100 s). Methods: T, trap; D, drift diving; O, observation; V, video; S, sonar. The superscript numerals indicate additional survey methods to the main category (Method) and are explained in the footnotes. In some tributaries only eDNA has been sampled (E) and years sampled are indicated by “X”, but in most, eDNA has been sampled in addition to other monitoring activities. The full list of eDNA sampling sites with results are presented in Figures 4, 5, 6. 0, monitored, no pink salmon; empty cell, not monitored.

^a^ Helicopter counts and drift diving.

^b^ Fishery catch.

^c^ Drift diving.

^d^ Drift diving/video.

For the total estimates of pink salmon entering the Teno River, the 2017 run size was simply estimated based on catch information and application of a general exploitation rate for one‐sea‐winter Atlantic salmon (Anon, 2019), which are of similar size to pink salmon. Annual sonar counting of all upstream migrating fish in the Teno main stem was started in 2018 in Polmak, in the lower part of the Teno main stem, c. 55 km from the estuary. In 2019, the pink salmon run was estimated based on sonar counts of fish <65 cm and species distribution in catches in this size class from the fishing zone near the sonar site (Anon., 2019; Pohjola et al., 2020). For 2021 and 2023 when more comprehensive video monitoring was established at the sonar site, Räty et al. (2025) provided a Bayesian modelling method for estimating the pink salmon run based on the sonar and video data (medians + probability intervals [PI]). Sonar monitoring has also been undertaken in recent years in the large headwater tributaries at Kárášjohka (2010, 2012, 2017–2024), Iešjohka (2019, 2020, 2022, 2024), Anárjohka (2018, 2019, 2021, 2023) and in two other tributaries in some years: Máskejohka (2020, 2022) and Veahčajohka (2016, 2021, 2024) (Table 1 and Figure 1).

Snorkelling counts of spawning Atlantic salmon (cf. Orell & Erkinaro, 2007) at three small tributaries, Buolbmátjohka, Njiljjohka and Áhkojohka (Table 1 and Figure 1) that cover central stretches of Atlantic salmon production areas of these rivers has been operational since 2003. Snorkelling counts in several other tributaries has also taken place in some earlier years, but not on a regular, annual basis (Table 1.)

In order to collate data on pink salmon occurrence, abundance and distribution, information on catches in local and tourist fisheries were compiled from postal questionnaires sent to fishermen, interviews and logbooks associated with annual estimation of Atlantic salmon catches (Erkinaro et al., 2019; Niemelä et al., 2005). More systematic such catch data have also been compiled since 1974, along with anecdotal catch records of pink salmon available since the early 1960s (e.g. Bjerknes & Vaag, 1980; Niemelä et al., 2016). Other sources of information used came from recent reports on an experimental pink salmon fishing project (Lukkari et al., 2024), helicopter surveys (Fagard, 2025; Muladal & Fagard, 2021) and visual observations of pink salmon occurrence in some tributaries (Table 1).

### 2.3 eDNA sampling and analyses

eDNA samples were collected once during August, during the peak spawning season of pink salmon (Erkinaro et al., 2022) from 19 localities in 2019 and 24 localities the other years (cf. Fossøy et al., 2022) across various tributaries but not the mainstem of the Teno River. Water was filtrated in duplicates per station on a 2.0 μm glassfiber filter (Merck Millipore) in 2019 and on a 0.8 μm capsule filter (Sterlitech GF 5.0/PES 0.8 μm) in the other years using a peristaltic pump (Bürkle Vampire). DNA was conserved by adding ATL‐buffer (Qiagen) and the filters were stored at room temperature until further analyses at the Centre for Biodiversity Genetics (NINAGEN) in Trondheim. DNA‐extraction took place within 1–5 months after sampling depending on the year.

The 2.0 μm glass fibre filters were stored in 5 mL tubes containing 4.05 mL of ATL‐buffer immediately after sampling and DNA‐extraction was initiated by adding 450 μL proteinase‐K before incubation at 56°C overnight. Approximately 1.5 mL of ATL‐buffer was added to the 0.8 μm capsule filters immediately after sampling and DNA extraction was initiated by adding 130 μL of proteinase‐K (diluted 1:10) to the capsules before incubation at 56°C overnight. DNA was extracted from both types of water filters using a combination of NucleoSpin Plant II (Machery‐Nagel) spin columns and Blood & Tissue buffers (Qiagen). DNA was eluted in 200 μL of preheated AE buffer and thereafter re‐eluted for maximising the DNA output.

Species‐specific genetic markers for pink salmon (Gargan et al., 2021) were analysed using digital droplet PCR (ddPCR). PCR reactions consisted of ddPCR™ Supermix for Probes (No dUTP, Bio‐Rad Laboratories), 0.9 μM forward and reverse primers, 0.25 μM of the probes, dH_2_O and 5 μL of DNA. To generate droplets, an AutoDG™ Instrument (Bio‐Rad Laboratories) was used, with subsequent PCR amplification in a Veriti™ 96‐Well Thermal Cycler (Applied Biosystems). The following thermal cycling conditions were used: an initial denaturation step at 95°C for 10 min, 40 cycles of denaturation at 95°C for 30 s, annealing and extension at 60°C for 1 min, a final step of denaturation at 98°C for 10 min and a final hold at 4°C. PCR plates were transferred to a QX200™ Droplet Reader (Bio‐Rad Laboratories) to automatically detect the fluorescent signal in the droplets. QuantaSoft software v.1.7.4 (Bio‐Rad Laboratories) was used to separate positive from negative droplets, according to manufacturer's instructions. To prevent presence of false positives, we conservatively set a limit of minimum three positive droplets for assessing a sample as positive (Dobnik et al., 2015).

### 2.4 Ethics statement

Most of the fish data used in this study have been collected by non‐intrusive observations or eDNA. Part of the data are catch statistics and estimates from ordinary recreational and traditional fisheries. The invasive pink salmon is subject to strong removal actions in Norwegian/Finnish rivers.

## 3 RESULTS

### 3.1 Catches and counts of pink salmon

**FIGURE 2:**
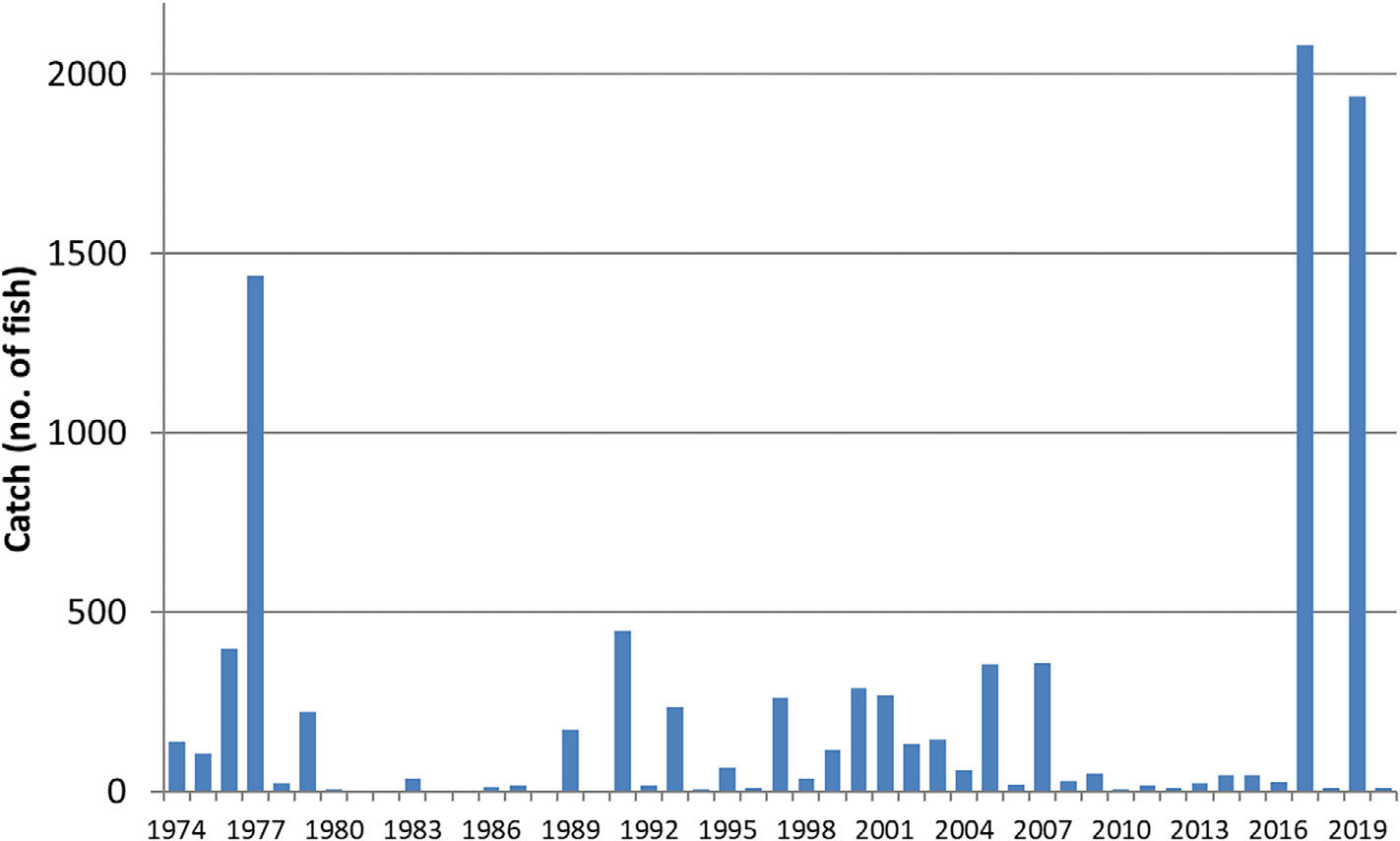
Estimated pink salmon catch (number of fish) in the Teno River catchment, 1974–2020.

Before 2017, estimated yearly catches of pink salmon in the Teno River have generally been low, varying within some tens or hundreds of individuals, with the exception of 1977, when more than 1400 pink salmon were caught (Figure 2.). In 2017 and 2019, the estimated pink salmon catches in the Teno River were c. 2000 fish, and since 2021 Atlantic salmon fishing in the river has been closed.

In the 1960s and 1970s, anecdotal catch reports from some tributaries of the Teno River provided the first indications of the spread of pink salmon in different parts of the catchment, including the headwater rivers Iešjohka, Kárášjohka and Anárjohka (Bjerknes & Vaag, 1980; Niemelä et al., 2016). In peak years of occurrence, e.g. 1960, 1965 and 1973, pink salmon have been captured far up in the system, up to 350 km distance from the sea (Bjerknes & Vaag, 1980).

**FIGURE 3:**
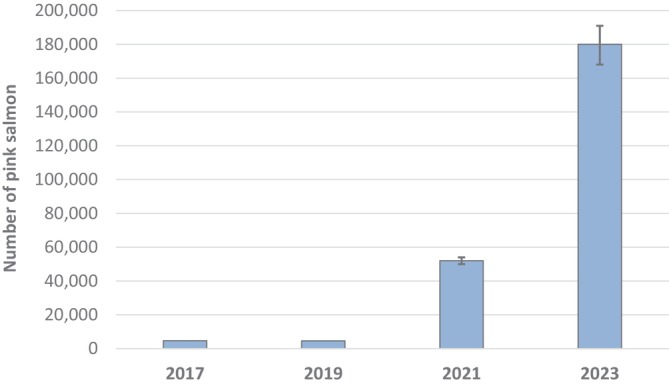
Estimated numbers of pink salmon which entered the Teno River in odd years 2017–2023. In 2019–2023 estimates are based on sonar counts in the lowermost part of the Teno main stem, the estimate in 2017 is based on catch data. Probability intervals for 2021 and 2023 estimates are based on methodology by Räty et al. (2025).

The numbers of pink salmon ascending the Teno River were estimated to be 4700 individuals in 2017, and a similar run size (4600) was estimated in 2019 at the sonar site in Polmak. The numbers of pink salmon entering the Teno system increased rapidly in 2021 and 2023, when an estimated 52,000 (90% PI 50000–54,000) and 180,000 (90% PI 168000–191,000) individuals passed the sonar site, respectively (Figure 3). These estimates do not include the pink salmon which migrated to, and remained at, the lower 55 km part of the Teno main stem and tributaries running to this area. For example, in 2023, a total of 2600 pink salmon were captured at the Máskejohka trap (Nasjonal kompetansegruppe for tiltak mot pukkellaks 2024), which is situated downstream from the Polmak sonar site. In addition, large schools (hundreds to >1000 individuals) of spawning pink salmon were observed in the lowermost part of the Teno mainstem downstream from the Polmak sonar site in 2021 and 2023 (Fagard, 2025; Muladal & Fagard, 2021), which add to the overall numbers of pink salmon that entered the Teno River.

In the early 2000s, small numbers of pink salmon were observed via video monitoring migrating to the Ohcejohka and Lákšjohka, tributaries of the Teno River, but no pink salmon have been observed in the snorkelling counts at the three small tributaries, Buolbmátjohka, Njiljjohka and Áhkojohka, which have been monitored since 2003 (Data presented since 2005 in Table 1). Pink salmon were first observed in Veahčajohka in 2016, and since 2017, records of increasing abundances in more tributaries have been made (Table 1). First counts and estimates of pink salmon runs reaching hundreds of individuals in single tributaries have been available since 2019 (Kárášjohka and Anárjohka) and especially in 2021 and 2023 (Table 1). In Ohcejohka, pink salmon abundance increased from a few fish until 2016 to 50 in 2017 and hundreds in 2021 and 2023. In Veahčajohka, three pink salmon were detected in 2016 and 380 in 2021 (Table 1). In 2023, more tributaries than before were surveyed by different methods, many by snorkelling. This revealed a widespread occurrence of pink salmon in numerous tributaries of the Teno River. In some tributaries the run sizes in 2023 were larger than in earlier years, with just over 2500 and 3800 pink salmon entering Máskejohka and Anárjohka, respectively (Table 1).

Based on helicopter surveys and snorkelling investigations, it appears evident that the largest spawning aggregations of pink salmon anywhere in the catchment have been in the middle reaches of the Teno main stem, despite the significant numbers of pink salmon entering the tributaries in recent years, especially in 2021 and 2023. Two large areas in the middle part of the main stem, downstream from the outlets of rivers Veahčajohka and Leavvajohka (Figure 1 and Table 1), included the main congregations of spawning pink salmon where several thousands to 30,000, and 10,000 to 50,000 individuals, respectively, were estimated on 30 July 2023 by a helicopter survey (Fagard, 2025). Similarly, large spawning aggregations of pink salmon have been observed by snorkelling in the Teno main stem in early August 2021 and 2023 in three separate areas located few kilometres up‐ and downstream from the River Ohcejohka outlet (Erkinaro et al., 2022; unpublished data). Finally, the experimental pink salmon fishery conducted in 2023 in the Teno main stem resulted in the largest catches at and close to the areas indicated by the observational methods described above (Lukkari et al., 2024).

### 3.2 eDNA detection and concentration

**FIGURE 4:**
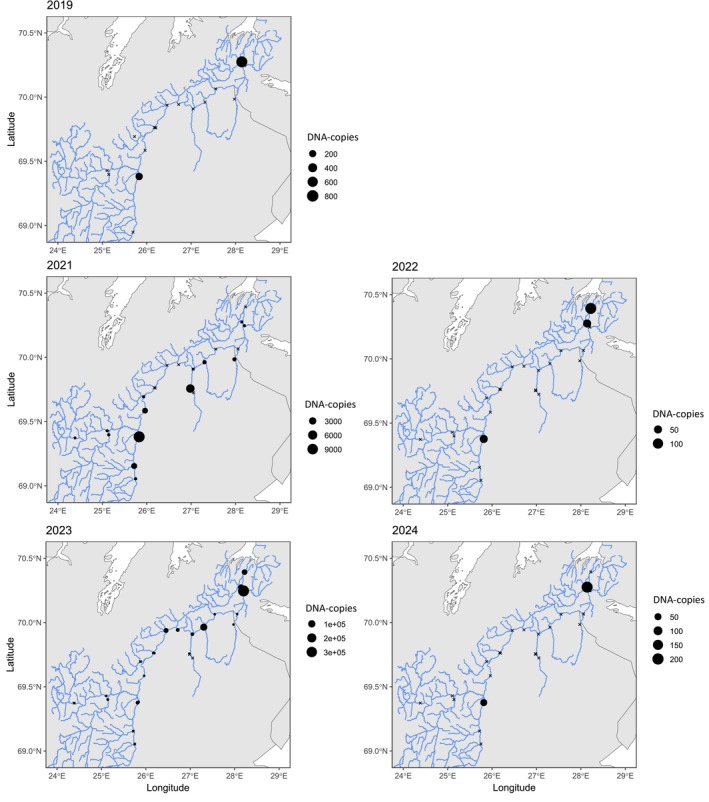
Spatial variation in detection and eDNA concentration of pink salmon for the different tributaries and localities in the Teno River. Note the different scales for DNA concentration among years and that each locality represents the mean of two samples. A cross indicates a negative detection and black dots positive detections. The legend symbols for DNA copies indicate discrete classes, e.g. in 2019, the smallest class is 1–200 copies, the next one 201–400 copies etc. See Figure 5, Table 1 and Figure 1 for locations.

**FIGURE 5:**
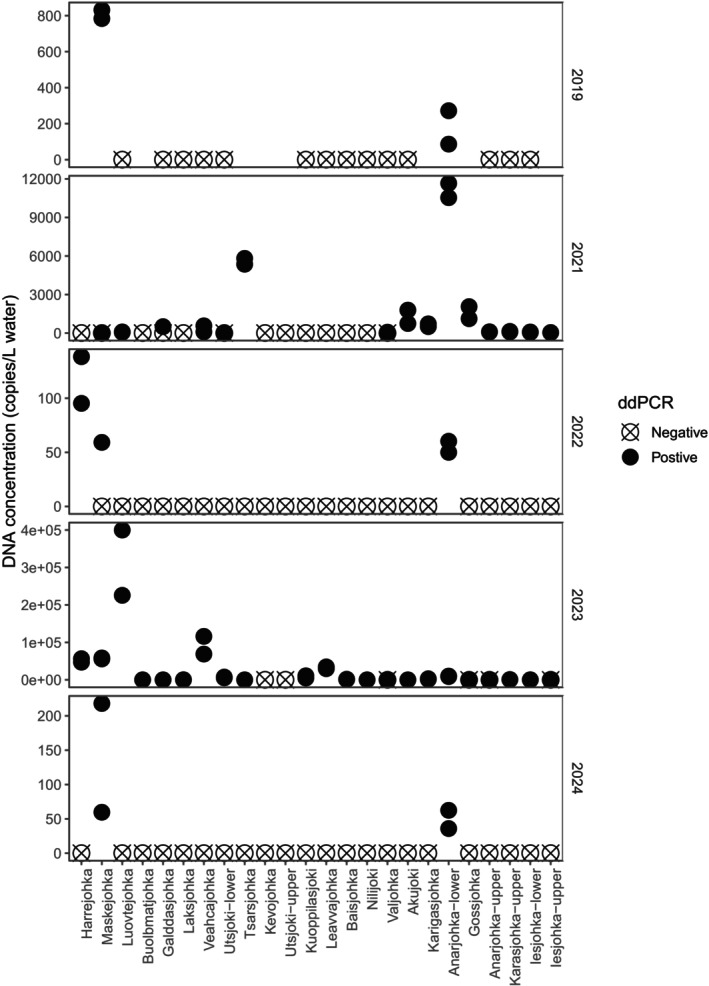
Detections of pink salmon by eDNA concentrations over years among tributaries of the Teno catchment. Localities are sorted by distance from the sea starting with downstream tributaries on the left and upstream tributaries on the right (see Table 1 and Figure 1 for locations). Each locality is represented by two different eDNA filters collected at the same time. Note the different scales on the *y* axes among panels.

The number of sites with eDNA detections of pink salmon showed large variation among both years and localities, and in particular between odd and even years as expected (Figures 4 and 5). There was a gradual increase in the proportion of detections in odd years, with two of 16 (12.5%) localities in 2019, 14 of 24 (58.3%) localities in 2021 and 22 of 24 (91.7%) localities in 2023 (Figure 3). In comparison, during even years pink salmon was only detected in three of 24 localities (12.5%) in 2022 and two of 24 localities in 2024 (8.3%) (Figures 4 and 5).

The spatial variation in both detections and DNA concentration among localities showed some similarities among years, where in particular the Anárjohka‐lower locality had consistent detections and also among the highest DNA concentrations every year, including the two even years (Figure 4). In addition, one of the three lowermost tributaries showed the highest concentration in all years, except for 2021, although the exact tributary changed among years.

The temporal variation in DNA concentration was large, with 2023 showing exceptionally high levels, particularly in the Luovtejohka tributary (Figure 5). Four other tributaries also had very high DNA concentrations in 2023: Hárrejohka and Máskejohka relatively close to Luovtejohka and the estuary of the Teno River, and Levvajohka and Veahčajohka in the mid‐part of the Teno catchment (Figure 5). Interestingly, the mid‐part of the Teno main stem also showed large spawning aggregations as observed by helicopter surveys and snorkelling investigations.

**FIGURE 6:**
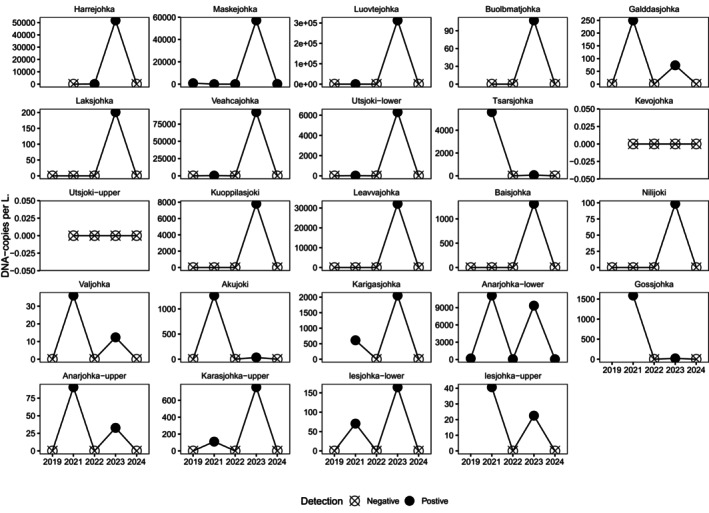
Temporal trends for pink salmon eDNA detections and concentrations (DNA copies per L) for individual tributaries in the Teno catchment. Localities are sorted by distance from sea starting with downstream tributaries on the top left and upstream tributaries on the bottom right, see Table 1 and Figure 1 for locations. Note the different scales on the *y* axes among panels.

When looking at each individual locality over time, most tributaries in the lower parts of the Teno catchment showed the same overall pattern, with 2023 having a much higher eDNA concentration than any other year (Figure 6). Interestingly, several tributaries showed relatively similar eDNA concentrations in 2021 and 2023, despite the more than three‐fold increase in pink salmon numbers estimated by sonar. This pattern seems to be most prevalent in tributaries of the upper parts of the Teno catchment, such as Váljohka, Anárjohka and Iešjohka (Figure 6). In two tributaries, Geavvu and Ohcejohka (upper part), the ddPCR analyses did not detect pink salmon in any year (Figure 6).

## 4 DISCUSSION

The diverse monitoring program for Atlantic salmon in different parts of the Teno River system has provided a unique opportunity for collecting long‐term information on pink salmon invasion, distribution and abundances across the mainstem and tributaries of this large catchment. The monitoring data gathered indicated a rapid increase in overall numbers of pink salmon since 2017 and their increasing distribution within the Teno system.

The recent development in pink salmon abundance in the Teno River has followed the general pattern observed in the Barents Sea area, across northern Norwegian rivers (Staveley, Ahlbeck‐Bergendahl, et al., 2025) as well as in those in northwest Russia (ICES, 2024). However, the observed decline in 2023 in the Russian White Sea and Archangelsk regions (NASCO, 2024) is a clear deviation from this. This decline raises a question about the potential directions of the future development of pink salmon abundance in the Barents Sea area in general. However, strong fluctuations in pink salmon population dynamics are also typical in their native Pacific area (e.g. Irvine et al., 2014; Krkošek et al., 2011).

Before the abrupt, steep increase in pink salmon abundances since 2017, a single outlying peak in pink salmon catch occurred in the Teno River in 1977. According to the yearly numbers of released juveniles in northwestern Russia published by Niemelä et al. (2016), the stocking effort behind the pink salmon year class returning in 1977 was relatively large with more than 5 million juveniles released in 1976, but this was not exceptional when compared to many other years in the 1960s and 1970s. It has been suggested that favourable conditions in the Barents Sea may have contributed to the overall high returns and catches of pink salmon in the mid‐1970s in the Kola Peninsula and White Sea area, and also in northern Norway (Hindar et al., 2020; Niemelä et al., 2016; Sandlund et al., 2019). Similarly, Atlantic salmon returns and catches were high in the Barents Sea rivers in the mid‐1970s, and linkages between salmon abundance and sea water temperatures have been suggested as explanatory factors (e.g. Niemelä et al., 2004; Pasanen et al., 2017).

The reasons behind the substantial increase in pink salmon abundance since 2017 are still unclear, but corresponding changes in the environment, and especially in oceanic conditions, have been proposed as likely candidates (Hindar et al., 2020; Lennox et al., 2023). Increased survival in the early marine phase of their life cycle is likely a major factor explaining the increasing size of pink salmon populations (Dunmall et al., 2025; Lennox et al., 2023), and spring temperatures along the northwest Russian and Norwegian coasts have indeed increased during the past couple of decades, with the marine habitat possibly becoming more suitable for the survival of young pink salmon (Dunmall et al., 2025).

The spread and distribution of pink salmon across different tributaries of the Teno system reflect certain patterns. First, it is evident from our data, and indicated already before 2017 (Niemelä et al., 2016), that pink salmon have entered the uppermost headwater tributaries of the Teno catchment, the large branches of Kárášjohka and Anárjohka, up to c. 250–350 km from the sea. Second, based on monitoring data, it seems that pink salmon first entered the largest tributaries (Veahčajohka, Ohcejohka, Kárášjohka and Anárjohka) and have only recently started to invade also smaller ones (Table 1). Third, there are observations indicating that in many cases, the largest spawning congregations of pink salmon have concentrated in the lowermost parts of the tributaries, for example in the Veahčajohka and Ohcejohka (unpublished monitoring data and personal observations).

The eDNA data collected from different tributaries indicated somewhat similar patterns with pink salmon observations in other monitoring programs: in 2019, pink salmon eDNA was detected predominately in large tributaries like the Máskejohka, Kárášjohka and Anárjohka, whereas more and more smaller tributaries showed detections in 2021 and especially in 2023 (Figures 4 and 5). Interestingly, there are some discrepancies between visual observations and the eDNA data. In particular, many snorkelling surveys in small tributaries have not resulted in pink salmon records, but eDNA detections have been evident in Buolbmátjohka, Njiljjohka and Áhkojohka in 2023, and in Áhkojohka in 2021 (Figures 4 and 5). It is possible that either the timing of the snorkelling surveys or their spatial coverage limited the probability of pink salmon detection during the snorkelling surveys. In many cases snorkelling surveys have been conducted in early to mid‐September, which is clearly after the main spawning season of pink salmon (Erkinaro et al., 2022). This could mean that potential pink spawners may have already died and flushed to the main stem. The pink salmon providing the eDNA signal may also have resided in the very lowermost parts of these small tributaries whereas the focus in many (e.g. Buolbmátjohka, Njiljjohka) snorkelling surveys have been the Atlantic salmon spawning areas further upstream.

Fagard (2025) reported data from helicopter surveys including locations of spawning aggregations of pink salmon along the Teno main stem in 2023. Based on these and earlier surveys (Muladal & Fagard, 2021) and snorkelling experiments (e.g. Erkinaro et al., 2022), it seems evident that the largest spawning aggregations of pink salmon are to be found in the main stem of the Teno River. According to Fagard (2025), the largest aggregations of pink salmon were detected on two localities in the central part of the Teno main stem, near the outlets of the tributaries Veahčajohka and Goahppelašjohka (see Figure 1 and Table 1 for location), c. 95–125 km from the estuary, at the beginning of the spawning period in 2023.

Some of the most abundant pink salmon aggregations in the main stem appear to be linked with high eDNA concentrations in tributaries running close to these main stem areas: two tributaries with high eDNA concentrations, Veahčajohka and Leavvajohka, are running to the main stem broadly in the same mid‐section of the Teno River where the two largest aggregations of spawning pink salmon were observed by Fagard (2025). In addition, the location of high spawner abundance observed in the upper part of the Teno main stem (Fagard, 2025) is close to the lower Anárjohka, which showed both high eDNA concentrations and runs of ascending fish in all years since 2019. However, the eDNA data suggests that overall, the largest congregations of pink salmon were found in the lower part of the Teno watercourse, as the highest DNA‐concentrations were found every year in one of the three lowermost tributaries, except for 2021 (Figure 6). In addition, the eDNA data suggest that the lower tributaries as well as the lower Anárjohka locality in the upper part of the catchment were preferred by the pink salmon, also in even years (Figures 4 and 5). Importantly, DNA‐concentrations are dependent on water flow and the size of each tributary, and these two factors will affect the eDNA results (see below). Unfortunately, eDNA has so far not been sampled from the main stem.

While there was a more than three‐fold increase in pink salmon numbers entering the Teno system as estimated by the sonar counts in the lower mainstem in 2023 compared to 2021, based both on fish counts and eDNA, the upper parts of the river system showed somewhat different results. For example, numbers of pink salmon in the Kárášjohka (Table 1) and eDNA concentrations in the Anárjohka and the upper Iešjohka were lower in 2023 than in 2021 (Figure 6). The highest concentrations ranging from thousands to the extreme values of hundreds of thousands of DNA copies per litre of water were all located in tributaries in the lower or mid‐part of the system, and all of these tributaries showed a marked increase in DNA concentrations from 2021 to 2023 (Figure 6) following the overall increase in pink salmon abundance in the Teno catchment. Hence, the eDNA data corroborate that the increased overall numbers of pink salmon in 2023 may largely have spawned in middle and lower parts of the watercourse (see also Fagard, 2025 and above).

The quantitative information extracted from eDNA data is still debated, but an increasing number of studies have found strong correlations with other types of data on fish numbers both when using species‐specific assays (e.g. Berger et al., 2024; Fauchet et al., 2025; Rourke et al., 2022) and DNA metabarcoding (e.g. Pont et al., 2023; Wu et al., 2024). eDNA concentrations depend on several environmental factors, such as river size, water flow and temperature, as well as fish size and activity patterns (Rourke et al., 2022). Interestingly, the pink salmon may be the perfect model species for extracting quantitative data as there is a single age class consisting of relatively homogenous individuals of similar size. With its strict 2‐year life cycle, there are no juveniles or other adults in the river before the pink salmon run begins in the summer/autumn. However, 10 fish in a small tributary will likely result in a much higher eDNA concentration than 10 fish in a big tributary, for example. Controlling for discharge has been shown to improve abundance estimates for migrating Atlantic salmon smolts (Fauchet et al., 2025). Unfortunately, we do not have discharge data for all the tributaries of the Teno catchment. Furthermore, our study only includes a single locality per tributary and a single sampling event per year. Including multiple localities and sampling events each year would potentially improve the abundance estimates. However, even with all these caveats, the eDNA data in general corroborate with, and extends the knowledge from, conventional monitoring methods used in this study.

In their recent paper, Staveley, Hellström, et al. (2025) highlighted the need and opportunity to use eDNA for detecting pink salmon in addition or together with conventional monitoring methods, citizen science and camera traps, especially in areas where pink salmon are still in low numbers. In the Teno catchment, the numbers of pink salmon have recently been very high in general, but for tributary‐specific monitoring in areas where no other monitoring activities are in place and/or where pink salmon abundance is not yet high, eDNA appears to be a practical and effective method for detecting the alien species.

Recent studies have documented strong impacts of pink salmon on both freshwater and marine ecosystems in the Pacific area (Ruggerone et al., 2023, 2025). In addition, marine‐derived nitrogen and phosphorus transported into inland freshwater systems and surrounding forest ecosystems by Pacific salmon has shown to strongly affect riparian productivity and plant diversity (Hocking & Reynolds, 2011; Kieran et al., 2021). In the Atlantic area, several risk assessments of possible ecosystem impacts have been made (Lennox et al., 2023; Staveley, Ahlbeck‐Bergendahl, et al., 2025), but information, data and published documentation on such impacts are lacking. Lennox et al. (2023) reviewed the potential harmful effects of pink salmon on native Atlantic migratory salmonid species in fresh water and listed the main risks as: impacts on behaviour of adult native salmonids during spawning migration, disturbance during spawning, effects of juveniles on space and food base of native species, impacts of nutrient loading from pink salmon carcasses, and overall reduced reproductive success of native salmonids and reduced value of recreational fisheries. In addition, Lennox et al. (2023) predicted possible impacts on native species in estuaries and nearby coastal areas, where pink salmon may locally occur in large numbers.

Successful reproduction of pink salmon, juvenile production and feeding of juveniles has been documented at the Teno River, in the main stem and in at least two tributaries (Erkinaro et al., 2022, 2024; Pohjanheimo, 2025; unpublished data). Active feeding of juvenile pink salmon may suggest potential for impact on the food base for juveniles of native salmonids, at least locally at a small spatial scale (Erkinaro et al., 2024). A recent study from two Barents Sea rivers, the Teno River and the nearby Näätämöjoki/Neidenelva River, on pathogens carried by pink salmon did not find evidence of particular risks of parasites and other pathogens to native fish species (Holopainen et al., 2025), although the study demonstrated that pink salmon have the potential to carry pathogens to native ecosystems.

Dunmall et al. (2025) listed the most important current gaps in information needed to prepare for a future development in pink salmon abundance and distribution in Atlantic regions, and identified needs to increase research efforts on pink salmon interactions with other salmonids and ecosystems, e.g. through pathogen spreading, behavioural impacts, trophic cascades and changes in water quality due to decomposition of pink salmon carcasses. Similarly, Staveley, Ahlbeck‐Bergendahl, et al. (2025) underscored the need to fill the current knowledge gaps, which are hampering the understanding of the consequences of pink salmon invasion in the Atlantic area.

For the future impacts of pink salmon on the Teno River ecosystem, a crucial question is to what extent the planned mitigation efforts will succeed to intercept pink salmon and remove them from the river (Domaas et al., 2024; Sandodden et al., 2024; Thorstad et al., 2025). If even higher numbers of pink salmon enter and distribute themselves in the Teno catchment than in 2023 (c. 180,000 individuals), further spread into smaller tributaries is possible, with unknown consequences and effects on the native fish species and ecosystems.

Although the strong focus in the Atlantic area has been on the rapid increase in abundance and distribution of pink salmon in odd years since 2017, there are some indications of increased abundance of pink salmon detected in even years as well. The Russian catch data from the White Sea and Barents Sea areas appear to indicate an increase over the past c. 10 years in even years (NASCO, 2024), and anecdotal information from some northern Norwegian rivers suggests the same trend is evident there. However, there is little evidence to support this within the Teno system, except for some indications at the headwater tributaries Kárášjohka and Anárjohka (Table 1). This potential development in even‐year pink salmon numbers highlights the need for Atlantic countries to consider designing monitoring programs and possible management and mitigation measures for pink salmon across all years (see also Lennox et al., 2023).

## AUTHOR CONTRIBUTIONS

J.E., P.O. and F.F. conceived the study. All authors contributed to sampling design and data collection. F.F. was in charge of analysing the eDNA data. J.E. and F.F. led the writing of the manuscript. All authors contributed critically to the drafts and gave final approval for publication.

## FUNDING INFORMATION

The monitoring programs at the Teno/Tana River catchment have been funded by the organisations and institutes of the authors: Natural Resources Institute Finland, Norwegian Institute for Nature Research, Tanavassdragets Fiskeforvaltning, Statsforvalteren i Troms og Finnmark, and by the national fishery authorities in Finland (Ministry of Agriculture and Forestry) and Norway (Norwegian Environment Agency).

## CONFLICT OF INTEREST STATEMENT

The authors have no financial or non‐financial interests to disclose.

## Data Availability

Most raw data are provided within the manuscript, and the more detailed eDNA data are available on request.
